# Multiple malignant epithelioid mesotheliomas of the liver and greater omentum: a case report and review of the literature

**DOI:** 10.1186/s40792-017-0342-y

**Published:** 2017-05-10

**Authors:** Koji Minami, Hiroshi Okumura, Kiyokazu Hiwatashi, Sumika Matsukita, Tetsuro Setoyama, Kota Minamimagari, Yusuke Tsuruta, Ichiro Kanetsuki, Yoshito Ogura, Shigeho Maenohara, Shoji Natsugoe

**Affiliations:** 1 Department of Surgery, Kagoshima Kouseiren Hospital, Tenpozan 22-25, Kagoshima, 890-0061 Japan; 2 Department of Pathology, Kagoshima Kouseiren Hospital, Tenpozan 22-25, Kagoshima, 890-0061 Japan; 3 Department of Radiology, Kagoshima Kouseiren Hospital, Tenpozan 22-25, Kagoshima, 890-0061 Japan; 4 Department of Digestive Surgery, Breast and Thyroid Surgery, Graduate School of Medical Sciences, Kagoshima University, Sakuragaoka 8-35-1, Kagoshima, 890-8520 Japan

**Keywords:** Multiple malignant mesothelioma, Liver, Greater omentum, Epithelioid type

## Abstract

**Background:**

Malignant mesothelioma commonly arises from the pleura, but can also arise from the peritoneum, pericardium, and tunica vaginalis testis. However, malignant mesothelioma of the liver is extremely rare and coexistence with malignant mesothelioma of the greater omentum has not been described in the literature. In this case report, we present a case of multiple malignant mesothelioma of the liver and greater omentum.

**Case presentation:**

A 36-year-old woman was admitted to our hospital for the evaluation of an elastic hard mass in the right upper abdomen. Abdominal contrast computed tomography showed a cystic mass measuring 13 × 14 × 11 cm in the right liver lobe with enhanced mural nodule. Abnormal accumulation was identified in the liver and lower abdominal area on ^18^F-fluorodeoxyglucose positron emission tomography. The patient underwent hepatectomy of the posterior segment and partial resection of the omentum. The final pathological diagnosis was low-grade multiple malignant epithelioid mesothelioma based on characteristic immunohistochemical findings. As of 6 months postoperatively, the patient has shown no disease recurrence.

**Conclusions:**

We present the first case of a 36-year-old woman with multiple malignant mesothelioma of the liver and greater omentum.

## Background

Malignant mesothelioma commonly arises from the mesothelial surfaces of the pleural cavities and is usually associated with inhalation of asbestos fibers [1]. In contrast, peritoneal malignant mesothelioma is a rare disease, and malignant mesothelioma arising in the liver is extremely rare [2–12]. Moreover, malignant mesothelioma of the greater omentum is also extremely rare [13, 14]. We present herein the case of a 36-year-old woman with multiple malignant mesothelioma of the liver and greater omentum.

## Case presentation

**Fig. 1:**
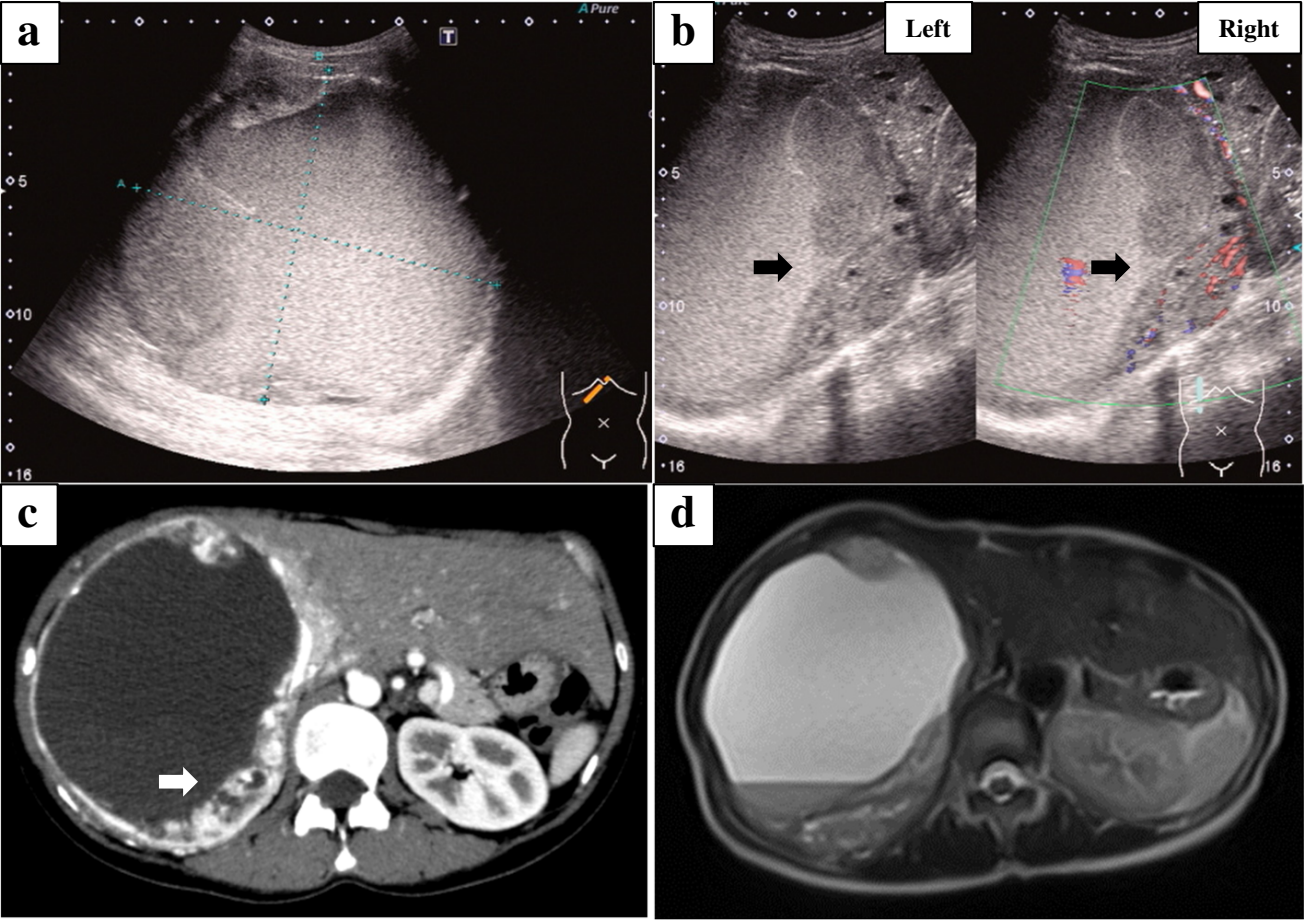
Findings from abdominal ultrasonography, CT, and MRI. **a** Abdominal ultrasonography shows an extensive space-occupying lesion in the right lobe of the liver, 15 cm in diameter. **b** *Left* photo shows a mural nodule, and *right* photo shows a heterogeneous internal component including hemorrhage and hypervascularity (*black arrow*). **c** Abdominal contrast-enhanced computed tomography shows a cystic mass measuring 13 × 14 × 11 cm in the right lobe of the liver with an enhanced mural nodule (*white arrow*). **d** Abdominal magnetic resonance imaging (MRI) shows a hyperintense component on T2-weighted imaging compatible with the hemorrhagic area

**Fig. 2:**
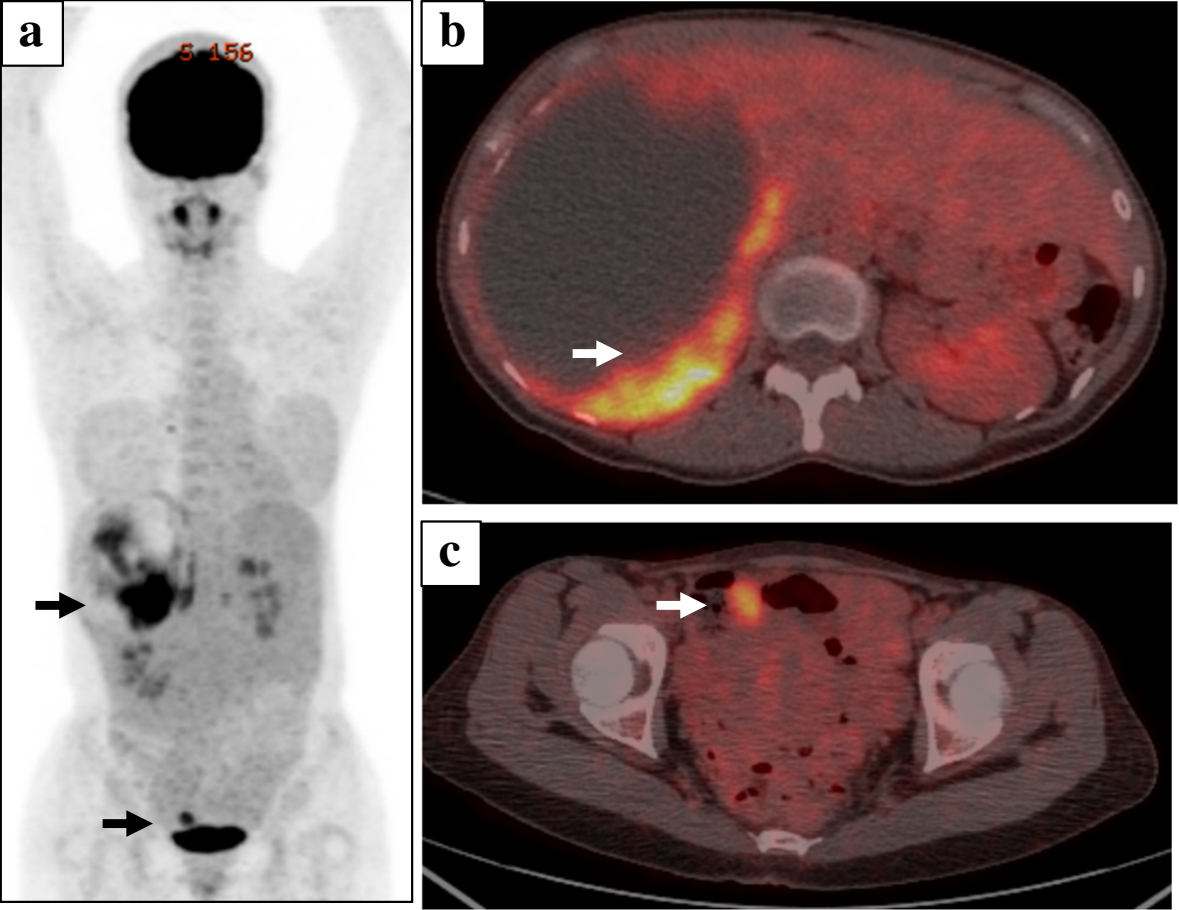
Findings of FDG-PET. **a** The fusion image of FDG-PET shows abnormal accumulation in the liver and lower abdomen (*black arrows*). **b** Cross-section of the upper abdomen indicates abnormal accumulation in the mural nodule in the liver (*white arrow*). **c** Cross-section of the lower abdomen indicates abnormal accumulation of the omental tumor (*white arrow*)

**Fig. 3:**
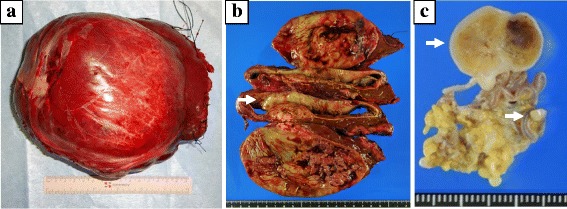
Gross findings of specimens. **a** The hepatic tumor represents a massive cystic tumor containing hemorrhagic fluid and measuring 18 × 15 cm. **b** The cut surfaces of the tumor show a mucinous or hemorrhagic, brownish or yellowish multinodular tumor in the extracystic wall area (*white arrow*). **c** The cut surfaces of the omental tumors indicate multiple solid, brownish, nodular tumors measuring 2.1 × 1.3 and 0.3 × 0.3 cm (*white arrows*)

**Fig. 4:**
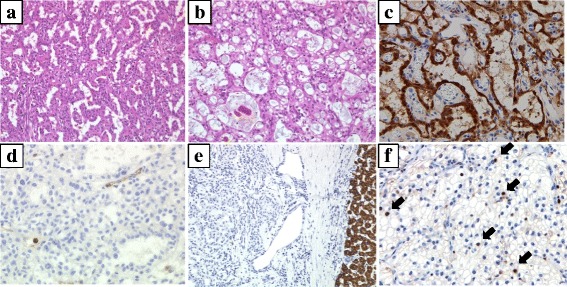
Histological findings of tumors. **a** Histological specimen of liver tumor (hematoxylin and eosin (HE), ×200) shows epithelioid-type mesothelioma cells with tubular components. **b** Mesothelioma cells with cystic components (HE, ×200). **c** Immunohistochemical staining for calretinin shows positive tumor cells (×200). **d** Immunohistochemical staining for CEA shows negative tumor cells (×200). **e** Immunohistochemical staining for HepPer1 shows negative tumor cells (×200). **f** Immunohistochemical staining for Ki-67, a marker of tumor proliferation, shows positive tumor cells (brown nuclei indicated with *black arrows*). Ki-67 index is 5–6%

A 36-year-old woman with a 2-month history of abdominal and back pain was referred and admitted to our hospital for treatment. The patient had no history of disease. On physical examination, a hard, elastic, poorly flexible mass was palpable in the right upper abdomen. No signs of obstructive jaundice or abdominal tenderness were identified. Laboratory analysis revealed anemia (hemoglobin = 10.6 g/dL), thrombocytosis (43.9 × 10^4^/μL), and elevation of C-reactive protein (2.71 mg/dl). The remaining laboratory examinations were within normal ranges, including tumor markers α-fetoprotein (AFP), carcinoembryonic antigen (CEA), and cancer antigen (CA)19-9. Abdominal ultrasonography revealed an extensive space-occupying lesion in the right lobe of the liver, 15 cm in diameter, showing a heterogeneous internal component including hemorrhage and hypervascular mural nodule (Fig. 1a, b). Contrast-enhanced computed tomography (CT) of the abdomen showed a cystic mass measuring 13 × 14 × 11 cm in the right lobe of the liver with enhanced mural nodule (Fig. 1c). Magnetic resonance imaging (MRI) of the abdomen showed hyperintense components on T2-weighted imaging, compatible with the hemorrhagic area (Fig. 1d). Abnormal accumulation was seen on ^18^F-fluorodeoxyglucose positron emission tomography (FDG-PET) of the liver and lower abdomen (Fig. 2). We then planned excision of the tumor and performed hepatectomy of the posterior segment and partial resection of the omentum, which had been detected on FDG-PET. Gross examination of the hepatic tumor showed a massive cystic tumor measuring 18 × 15 cm containing hemorrhagic fluid, and mucinous, hemorrhagic brownish or yellowish multinodular tumors were observed in the extra cystic wall area (Fig. 3a, b). The omental tumors were two slightly brownish, nodular tumors measuring 2.1 × 1.3 and 0.3 × 0.3 cm (Fig. 3c). Microscopically, both hepatic and omental tumors comprised tubular, cystic, or spindle structures of epithelioid cells with clear or eosinophilic cytoplasm (Fig. 4a, b). Immunohistochemically, tumor cells were positive for AE1/AE3, EMA, CK19, CK7, CD10, and calretinin (Fig. 4c), partly or weakly positive for CK5/6, D2-40, vimentin, and WT-1, and negative for HepPer1, chromogranin A, synaptophysin, CEA, inhibin α, MUC1, melan A, HMB45, CA19-9, ER, PgR, CD34, bcl-2, and β-catenin (Fig. 4d, e). Ki-67 index was 5–6% (Fig. 4f). The final pathological diagnosis was multiple low-grade malignant epithelioid mesothelioma. As of the time of writing, 6 months postoperatively, the patient has shown no disease recurrence.

### Discussion

Malignant mesothelioma commonly arises from the pleura, but can also arise from the peritoneum, pericardium, and tunica vaginalis testis [2]. However, malignant mesothelioma of the liver is extremely rare and coexistence with malignant mesothelioma of the greater omentum has not been addressed in the literature. Mesothelioma of the liver may arise from Glisson’s capsule, the hepatic falciform ligament or fibrous connective tissue, and then expand to the liver parenchyma [3]. To search the literatures, we used key words of intrahepatic, malignant, and mesothelioma and found 12 case reports which have described primary intrahepatic malignant mesothelioma [2–12] (Table 1). In detail, these cases involved six men and six women, with a mean age of 58.4 years (range, 41–68 years). Our case represents the youngest female case among these reported cases. Although conventional mesothelioma is frequently associated with asbestos exposure, only 1 case had a clear history of asbestos exposure. Among the remaining 10 cases, 8 cases had no history of asbestos exposure and 3 were not evaluated. One case had a history of viral hepatitis, and 9 cases did not. Mean tumor size was 12.2 cm (range, 3.2–24 cm), and our case showed the third largest mass. Gross finding of the tumor was a cystic mass which is not common, and there was no report of case with cystic tumor ever. This tumor contained hemorrhagic fluid; therefore, we thought that the tumor might have bled and formed cystic mass. In 10 cases, the tumor arose in the right lobe, as in our case, and only one tumor arose in the left lobe. Surgery was performed in 10 cases, and only 1 case received best supportive care, due to systemic lymph node swelling and rupture of the tumor [2]. Pathologically, malignant mesothelioma is classifiable into three subtypes: epithelioid, sarcomatoid, and biphasic. The epithelioid type is the most common type, and tubulopapillary or solid variations can be seen in the tumor [3]. The tumor in our case was also diagnosed as epithelioid type, but showed atypical findings such as solid and tubular, cystic, or spindle components in the tumor, clearly partitioned from normal liver tissue by a fibrous capsule. The tumor showed partial hyalinization, but no necrosis. Typical immunohistochemical features are positive results for calretinin, vimentin, cytokeratin, WT-1, and D2-40 and negative results for CD34, CEA, AFP, and Ber-EP4, as seen in our case [3]. In terms of tumor proliferative activity, Ki-67 index in the typical malignant mesothelioma is 15–20% [11], but was 5–6% in our case. The tumor was therefore diagnosed as a low-grade malignant tumor, and metastasis was considered unlikely, although a primary malignant omental mesothelioma is also a rare disease. We found only 2 case reports of malignant mesothelioma of the omentum researching with key words of greater omentum, malignant, and mesothelioma [13, 14]. It was difficult to distinguish multiple tumor from metastatic omental tumor in our case. Multiple malignant mesotheliomas and metastasis of low-grade malignant mesothelioma are both unlikely. The omental tumor cells were positive for AE1/AE3, CK19, CK7, EMA, C D10, and calretinin, partly for CK5/6, D2-40, and vimentin, and negative for HepPer1, chromogranin A, synaptophysin, CEA, inhibin, MUC1, melan A, HMB45, CA19-9, ER, PgR, CD34, bcl-2, and β-catenin that was same findings with hepatic tumor. However, both tumors have fibrous capsule without invasion of tumor cells. Moreover, both tumors had lower proliferated activity and considered to be low-grade malignant tumor. Basing on these pathological findings, we should diagnose the tumors as multiple mesotheliomas, although we are not able to deny a possibility of dissemination. Concerning about the outcome, lymph node relapse has only been reported in 2 cases, and they were alive at 2 or 36 months after relapse without hematogenous metastatic disease [6, 9]. Our patient remains alive as of 6 months after surgery without relapse.

**Table 1:** Summary of hepatic mesothelioma

Categories	Data (*n* = 12)
Mean age (range)	58.4 years (41–68 years)
Male/female	6/6
Asbestos exposure	
(+/−/NE)	1/8/3
Viral hepatitis	
(+/−/NE)	1 (C type)/9/2
Mean tumor size (range)	12.2 cm (3.2–24 cm)
Location	
(Right/left/NE)	10/1/1
Treatment	
(Surgery/BSC/NE)	10/1/1
Pathological type	
(Epithelioid/sarcomatoid/biphasic)	9/0/3
Relapse	
(+/−/NE)	2 (Lymph node)/5/5

*NE* not evaluated; *BSC* best supportive care

## Conclusions

We presented the case of a 36-year-old woman with multiple malignant mesothelioma of the liver and greater omentum with review of the literatures.

## References

[1] Zellos L, Christiani DC (2004). Epidemiology, biologic behavior, and natural history of mesothelioma. Thorac Surg Clin.

[2] Inagaki N, Kibata K, Tamaki T, Shimizu T, Nomura S (2013). Primary intrahepatic malignant mesothelioma with multiple lymphadenopathies due to non-tuberculous mycobacteria: a case report and review of the literature. Oncol Lett.

[3] Serter A, Buyukpinarbasili N, Karatepe O, Kocakoc E (2015). An unusual liver mass: primary malignant mesothelioma of the liver: CT and MRI findings and literature review. Jpn J Radiol.

[4] Imura J, Ichikawa K, Takeda J, Iwasaki Y, Tomita S, Kubota K (2002). Localized malignant mesothelioma of the epithelial type occurring as a primary hepatic neoplasm: a case report with review of the literature. APMIS.

[5] Leonardou P, Semelka RC, Kanematsu M, Braga L, Woosley JT (2003). Primary malignant mesothelioma of the liver: MR imaging findings. Magn Reson Imaging.

[6] Gutgemann I, Standop J, Fischer HP (2006). Primary intrahepatic malignant mesothelioma of epithelioid type. Virchows Arch.

[7] Kim DS, Lee SG, Jun SY, Kim KW, Ha TY, Kim KK (2008). Primary malignant mesothelioma developed in liver. Hepatogastroenterology.

[8] Sasaki M, Araki I, Yasui T, Kinoshita M, Itatsu K, Nojima T (2009). Primary localized malignant biphasic mesothelioma of the liver in a patient with asbestosis. World J Gastroenterol.

[9] Buchholz BM, Gutgemann I, Fischer HP, Gorschluter M, Turler A, Kalff JC (2009). Lymph node dissection in primary intrahepatic malignant mesothelioma: case report and implications for diagnosis and therapy. Langenbecks Arch Surg.

[10] Dong A, Dong H, Zuo C (2014). Multiple primary hepatic malignant mesotheliomas mimicking cystadenocarcinomas on enhanced CT and FDG PET/CT. Clin Nucl Med.

[11] Perysinakis I, Nixon AM, Spyridakis I, Kakiopoulos G, Zorzos C, Margaris I (2014). Primary intrahepatic malignant epithelioid mesothelioma. Int J Surg Case Rep.

[12] Haji Ali R, Khalife M, El Nounou G, Zuhri Yafi R, Nassar H, Aidibe Z (2017). Giant primary malignant mesothelioma of the liver: a case report. Int J Surg Case Rep.

[13] Shin MK, Lee OJ, Ha CY, Min HJ, Kim TH (2009). Malignant mesothelioma of the greater omentum mimicking omental infarction: a case report. World J Gastroenterol.

[14] Liu YC, Kuo YL, Yu CP, Wu HS, Yu JC, Chen CJ (2004). Primary malignant mesothelioma of the greater omentum: report of a case. Surg Today.

